# Therapeutic functions of tanshinone IIA in a zebrafish model of glucocorticoid-induced osteoporosis

**DOI:** 10.3389/fphar.2026.1772925

**Published:** 2026-03-18

**Authors:** Xinyu Li, Xiaoyang Zhou, Zhiyong Li, Zilu Zhu, Songtao Wang, Jiaolong Huang, Kai Lian, Peng Duan, Chunhui Hu, Yihua Shi

**Affiliations:** 1 Department of Orthopedics, Xiangyang No. 1 People’s Hospital, Hubei University of Medicine, Xiangyang, China; 2 Key Laboratory of Zebrafish Modeling and Drug Screening for Human Diseases of Xiangyang City, Xiangyang No. 1 People’s Hospital, Hubei University of Medicine, Xiangyang, China; 3 Department of Orthopedics, Postgraduate Union training base of Xiangyang No. 1 People’s Hospital, School of Medicine, Wuhan University of Science and Technology, Xiangyang, China; 4 Department of Ophthalmology, Xiangyang No. 1 People’s Hospital, Hubei University of Medicine, Xiangyang, China

**Keywords:** bone mineralization, glucocorticoid-induced osteoporosis, skeletal development, tanshinone IIA, transcriptomics, zebrafish

## Abstract

**Background:**

Tanshinone IIA (TSN), a compound extracted from *Salvia miltiorrhiza*, has demonstrated a range of pharmacological activities. However, its therapeutic efficacy in glucocorticoid-induced osteoporosis (GIOP) remains insufficiently understood. This study was performed to investigate the protective effects of TSN against prednisolone (PN)-induced osteoporosis in zebrafish larvae.

**Methods:**

The osteoprotective properties of TSN were assessed using alizarin red S staining, calcein staining, and alcian blue staining to evaluate bone mineralization, density, and cartilage development. Cardiovascular function and locomotor behavior were analyzed using transgenic zebrafish models and behavioral tracking systems. RNA sequencing was performed to identify differentially expressed genes and key signaling pathways. Protein-protein interaction networks were constructed to elucidate gene associations, and results were validated via quantitative reverse transcription polymerase chain reaction.

**Results:**

TSN administration effectively attenuated PN-induced developmental toxicity, enhanced bone mineralization and density, and improved cartilage abnormalities in zebrafish larvae. Transcriptomic analysis identified 505 genes with reversed expression profiles between the TSN and PN treatment groups, primarily associated with skeletal system development, lipid metabolism, and fatty acid β-oxidation. Key affected pathways included PPAR signaling, lipid atherosclerosis, and reactive oxygen species. Notably, skeletal development was characterized by the upregulation of genes including *col1a1b*, *col2a1b*, *col9a3*, *rdh1*, and *acana*.

**Conclusion:**

These findings demonstrate the osteoprotective effects of TSN in mitigating glucocorticoid-induced bone loss and cartilage abnormalities, highlighting its potential as a therapeutic agent for GIOP. The transcriptomic insights provide novel perspectives on TSN’s role in regulating bone metabolism and skeletal development.

## Graphical Abstract

**Figure:**
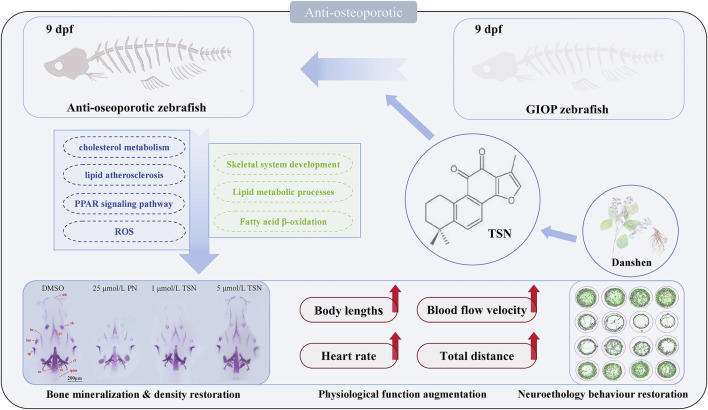

## Highlights

- TSN reduced PN-induced osteoporosis and chondrodysplasia in zebrafish larvae.
- TSN treatment influenced biological processes linked to skeletal development, lipid metabolic process and fatty acid β-oxidation.
- TSN alleviated osteoporosis via cholesterol metabolism, PPAR signaling, lipid atherosclerosis, and ROS.

## 1 Introduction

Osteoporosis is a systemic skeletal disorder characterized by reduced bone mass and the deterioration of bone tissue microarchitecture, ultimately leading to diminished bone strength and an increased risk of fractures (Chapurlat et al., 2020). Glucocorticoids (GCs), a class of steroid hormones synthesized by the adrenal cortex, play a vital role in regulating various physiological processes, including metabolism, immune function, and inflammatory responses (Timmermans et al., 2019). Although GCs are commonly used in the treatment of inflammatory conditions such as rheumatoid arthritis and systemic lupus erythematosus, their prolonged use is associated with several adverse effects—most notably, glucocorticoid-induced osteoporosis (GIOP) (Steinbuch et al., 2004). Epidemiological studies have consistently shown that the incidence of osteoporosis is significantly higher in patients undergoing long-term GC therapy (Chiodini et al., 2020).

The pathogenesis of GIOP involves a complex interplay of mechanisms that disrupt the delicate balance between bone resorption and formation. GCs exert their detrimental effects by inhibiting osteoblast activity, which is essential for bone formation, while simultaneously promoting osteoclast activity, thereby increasing bone resorption (Weinstein, 2012). This imbalance ultimately leads to bone loss and the development of GIOP (Lane, 2019; Chen et al., 2023). Given that an estimated 200 million individuals worldwide are affected by osteoporosis—and in light of a rapidly aging global population—the prevention and management of GIOP in patients requiring long-term GC therapy has become a significant public health concern (Zhang et al., 2024).

Current therapeutic strategies for GIOP include bisphosphonates, RANKL inhibitors, selective estrogen receptor modulators, and parathyroid hormone analogs (Peng et al., 2021). However, these treatments are associated with various side effects and limitations. For instance, bisphosphonates can cause gastrointestinal disturbances, hypocalcemia, and, in rare cases, severe adverse events such as osteonecrosis of the jaw (Lewiecki, 2010; Ganesan et al., 2024). Denosumab, a monoclonal antibody against RANKL, has recently emerged as a promising treatment for GIOP; however, concerns have been raised about its potential adverse effects on the cardiovascular system, central nervous system, and gastrointestinal tract (Lu et al., 2023; Hildebrand et al., 2024). Given these limitations, there is an urgent need to explore safer and more effective alternatives for the prevention and treatment of GIOP.

*Salvia miltiorrhiza* Bunge, commonly referred to as “danshen” or “red sage,” is a traditional Chinese herbal medicine with more than 2,000 years of documented use for treating various ailments (Kum et al., 2021). Recent research suggests that *S. miltiorrhiza* and its active constituents—particularly tanshinone IIA (TSN)—may have beneficial effects on bone metabolism in the context of osteoporosis (Guo et al., 2014; Wang et al., 2017; Ma et al., 2023). TSN, a lipophilic diterpene quinone, exhibits a broad spectrum of pharmacological properties, including anti-inflammatory, antioxidant, and neuroprotective effects (Li et al., 2015). Although previous studies have examined TSN’s potential in treating osteoporosis, its specific role in preventing or mitigating GIOP remains largely unexplored (Yang et al., 2019). This study found that tanshinone IIA has bone-protective effects. It acts through multiple targets and pathways to prevent osteoporosis. The compound promotes the differentiation of osteoblasts and accelerates bone formation. In addition, it improves the bone marrow microenvironment through its strong antioxidant properties. These features indicate its unique therapeutic potential. Etidronate disodium (ED), a first-generation bisphosphonate, regulates bone metabolism by inducing osteoclast apoptosis and supporting osteoblast function. Because of its well-established anti-resorptive effect and classic role in osteoporosis treatment, ED was used as a positive control to evaluate the therapeutic effect of TSN in managing GIOP (Queiroz Júnior et al., 2022). We determined the appropriate concentration of the positive control drug by reviewing previous studies on etidronate disodium intervention in GIOP. Literature data showed that a dose of 15 μg/mL effectively reduced GIOP-related pathological indicators (Zhou et al., 2024). This dose was also confirmed to be effective in our research group’s prior related experiments. Therefore, we selected 15 μg/mL as the positive control concentration for this study (Fan et al., 2025).

The zebrafish (*Danio rerio*) has become a prominent model organism for investigating human diseases, including skeletal disorders, because of its substantial genetic similarity to humans, rapid developmental processes, and optical transparency (Zuo et al., 2024). The zebrafish genome contains orthologues for approximately 82% of human disease-associated genes, making them an exceptional platform for drug discovery and toxicological research (Howe et al., 2013). This study aims to leverage the advantages of the zebrafish model to investigate the protective effects of TSN on prednisolone (PN)-induced osteoporosis and chondrodysplasia in larval zebrafish, while also elucidating the underlying molecular mechanisms through transcriptome analysis. The findings of this study will provide valuable insights into the potential of TSN as a novel therapeutic agent for GIOP and contribute to the development of safer and more effective treatment strategies for this debilitating condition.

## 2 Materials and methods

### 2.1 Chemicals and regents

TSN (CAS 568–72–9, 98% purity), ED (CAS 7414–83–7), and calcein (CAS 154071–48–4) were purchased from Aladdin Co., Ltd. (Shanghai, China). PN (CAS 83–43–2) was obtained from Adamas-beta (Shanghai, China). PN and ED were dissolved in dimethyl sulfoxide (DMSO). Potassium hydroxide (KOH) was acquired from Sigma (St. Louis, MO, United States), hydrogen peroxide (H2O2) from Tianli (Tianjin, China), and 4% paraformaldehyde from Beyotime Co., Ltd. (Shanghai, China). Alizarin red (CAS 130–22–3) was purchased from Beyotime Co., Ltd., and glycerol from Solarbio (Beijing, China). All experimental procedures were approved by the Committee for Animal Experimentation of Xiangyang No. 1 People’s Hospital, Hubei University of Medicine (Approval No. XYYYE20250031).

### 2.2 Animals

Adult wild-type AB zebrafish (*Danio rerio*) and flk:EGFP transgenic zebrafish were maintained in a recirculating aquaculture system under standard conditions (14 h light/10 h dark cycle, pH 7.0 ± 1.0, temperature 28 °C ± 0.5 °C). The fish were fed twice daily with newly hatched brine shrimp. Flk:EGFP transgenic zebrafish express enhanced green fluorescent protein (EGFP) in endothelial cells, enabling visualization of blood vessel development.

For embryo collection, mature zebrafish (3–4 months old) were placed in breeding tanks at a female-to-male ratio of 1:2 and kept separated overnight. The following morning, dividers were removed to allow spawning. Fertilized eggs were collected within 30 min of spawning and incubated in 10-cm petri dishes containing 50 mL of E3 medium (5 mM NaCl, 0.17 mM KCl, 0.33 mM CaCl2, 0.33 mM MgSO4) at 28 °C.

### 2.3 Experimental procedures

To determine the optimal PN concentration for inducing osteoporosis, a concentration gradient of PN (5, 10, 25, and 50 μmol/L) was used. PN was dissolved in 0.5% DMSO to prepare a 5 mmol/L stock solution, which was further diluted in E3 medium to achieve the desired concentrations. The control group was exposed to E3 medium containing 0.5% DMSO. Furthermore, TSN stock solution (800 μmol/L) was prepared in DMSO. For experimental dosing, the stock was diluted to ensure a final DMSO concentration of 0.5% across all groups, including the control.

For the main experiment, zebrafish larvae at 4 days post-fertilization (dpf) were randomly divided into six groups (n = 20 larvae per group) and exposed to the following treatments for 5 days: control (E3 medium containing 0.5% DMSO), PN (25 μmol/L PN), PN + ED (25 μmol/L PN + 15 μg/mL ED (Zhou et al., 2024; Fan et al., 2025), positive control), PN + TSN (1 μmol/L) (25 μmol/L PN + 1 μmol/L TSN), PN + TSN (5 μmol/L) (25 μmol/L PN + 5 μmol/L TSN), and PN + TSN (10 μmol/L) (25 μmol/L PN + 10 μmol/L TSN).

**FIGURE 1:**
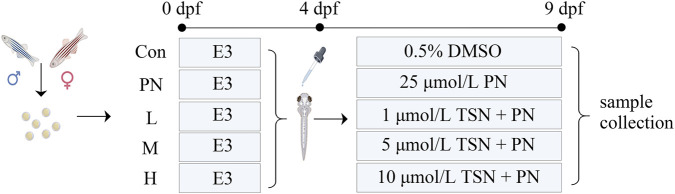
Schematic representation of the experimental animal study design. Con: normal control group, PN: osteoporosis model group, L: low dose group, M: medium dose group, and H: high dose group.

Treatments were conducted in six-well plates with 8 mL of solution per well. Media were renewed daily by replacing half of the volume. The entire investigation was carried out in accordance with the study protocol depicted in Figure 1, with all procedures adhering strictly to established ethical guidelines.

### 2.4 Alizarin red staining

Alizarin red staining was performed to assess cranial bone mineralization in zebrafish larvae. Zebrafish larvae for 9 dpf from each group (n = 10) were fixed overnight in 4% paraformaldehyde at room temperature. The fixed larvae were then bleached in a solution containing 3% H2O2 and 2% KOH for 8 h, until they became translucent. After removing the bleaching solution, the larvae were stained with 0.005% alizarin red S solution for 12 h. Excess stain was removed by washing the larvae in a graded series of glycerol/KOH solutions (3:1, 1:1, and 1:3) for 10 min each. The stained larvae were imaged using a stereomicroscope (Olympus, Tokyo, Japan), and the images were analyzed using Image-Pro Plus 6.0 software (Media Cybernetics, Rockville, MD, United States).

### 2.5 Calcein staining

Calcein staining was performed to assess bone mineralization in zebrafish larvae. Larvae for 9 dpf from each group (n = 10) were immersed in 0.2% calcein solution for 20 min, then washed three times (5 min each) with E3 medium until the eluate was clear. The larvae were anesthetized with 0.02% MS-222 (tricaine methanesulfonate), mounted in 3% methylcellulose, and imaged laterally using a fluorescence microscope (Guangzhou, China). The fluorescence intensity of the first three vertebrae (V1–V3) was quantified using ImageJ software (National Institutes of Health, Bethesda, MD, United States).

### 2.6 Alcian blue staining

Alcian blue staining was used to visualize cartilage development in zebrafish larvae. Larvae for 9 dpf from each group (n = 15) were fixed overnight in 4% paraformaldehyde at 4 °C. The fixed larvae were dehydrated in 75% ethanol for 20 min, then stained overnight with 0.1% alcian blue solution prepared in 70% ethanol containing 0.37% HCl. The stained larvae were rehydrated in a graded series of ethanol solutions (75%, 50%, and 25%) for 10 min each, followed by bleaching in a solution containing 3% H2O2 and 2% KOH for 6 h. Larvae were then cleared in a graded series of glycerol/KOH solutions (1:3, 1:1, and 3:1) for 10 min each and stored in 80% glycerol until imaging. The stained larvae were imaged ventrally using a stereomicroscope (Olympus), and the Meckel’s cartilage–palatoquadrate (M-PQ) angle was measured using ImageJ software.

### 2.7 Oil Red O staining

Zebrafish larvae at 9 dpf from each group (n = 6)were fixed overnight in 4% paraformaldehyde at room temperature. Following fixation, the larvae were washed three times with phosphate-buffered saline and then incubated in 60% isopropanol for 30 min to facilitate penetration of the staining solution. The larvae were stained with Oil Red O solution, prepared in 60% isopropanol, for 2.5 h to visualize neutral lipids and lipid accumulation. After staining, the larvae were washed with 60% isopropanol to remove excess stain and rinsed three times with phosphate-buffered saline to ensure clarity. Images of the stained larvae were captured using a stereomicroscope (Olympus) to assess lipid distribution and accumulation across different treatment groups.

### 2.8 Locomotor behavior analysis of larval zebrafish

The locomotor activity of zebrafish larvae was assessed at 9 dpf using a ZebraBox tracking system (ViewPoint, Lyon, France). Larvae from each group (n = 8) were individually placed in 48-well plates containing 2 mL of E3 medium per well. After a 30-min adaptation period, swimming activity was recorded for 30 min under alternating light and dark conditions (5 min each). The total distance traveled and the mean locomotor activity were analyzed using ZebraLab software (ViewPoint).

### 2.9 Oxidative stress analysis

Reactive oxygen species (ROS) production was measured using 2′,7′–dichlorofluorescin diacetate (DCFH–DA). The larvae were exposed to DMSO, TSN + PN and PN for 5 days. After exposure, we added a 1:1000 dilution of DCFH–DA solution to the treated larvae. The samples were then incubated in the dark at 28.5 °C for 40 min. After staining, we rinsed the larvae three times with MilliQ water. We observed the stained larvae using a fluorescence stereomicroscope (Olympus, Tokyo, Japan). We captured images and analyzed them with ImageJ software (National Institutes of Health, Bethesda, MD, United States). Each concentration included three replicates, with six larvae per replicate.

At 9 dpf, we randomly collected and homogenized zebrafish after TSN and PN exposure. We homogenized approximately 50 larval zebrafish in 400 μL of 0.9% NaCl. The homogenate was then centrifuged at 4000 rpm for 10 min at 4 °C. We collected the supernatant to measure malondialdehyde (MDA) and catalase (CAT) levels. We used assay kits from AIDISHENG Institute of Biotechnology (Jiangsu, China) and followed the manufacturer’s instructions. We determined the protein concentration in the supernatant using a BCA protein quantification kit (Beyotime Biotechnology Co., Ltd., Shanghai, China).

### 2.10 RNA sequencing (RNA-seq) analysis

For transcriptome analysis, nine zebrafish larvae of each group at 9 dpf (N = 3 replicates) were obtained and immediately transported to Huada Gene Technology Service Co., Ltd. (Wuhan, China) for RNA extraction and purification. RNA quality and quantity were assessed using a NanoDrop 2000 spectrophotometer (Thermo Fisher Scientific) and an Agilent 2100 Bioanalyzer (Agilent Technologies, Santa Clara, CA, United States). RNA samples with an RNA integrity number of ≥8.0 were used for library preparation and sequencing (Supplementary Table S1). The RNA-seq samples were not pooled. Each biological replicate was processed independently.

RNA-seq was performed by Huada Gene Technology Service Co., Ltd. using an Illumina HiSeq 4000 platform (Illumina, San Diego, CA, United States) with a paired-end 150-bp sequencing strategy. Raw reads were filtered to remove adaptor sequences, low-quality reads, and reads containing poly-N sequences. Clean reads were aligned to the zebrafish reference genome (GRCz11) using HISAT2 (version 2.1.0).

Differentially expressed genes (DEGs) were identified using the DESeq2 package (version 1.24.0) in R (version 3.6.1), with a threshold of |log2 (fold change)| >1 and adjusted P-value of <0.05, where the adjusted P-value (false discovery rate, FDR) was calculated using the Benjamini–Hochberg procedure. Gene Ontology (GO) and Kyoto Encyclopedia of Genes and Genomes (KEGG) pathway enrichment analyses were performed using the clusterProfiler package (version 3.12.0) in R. Protein–protein interaction networks (PPINs) were constructed using the STRING database (version 11.0) and visualized with Cytoscape software (version 3.7.2).

### 2.11 Quantitative real-time polymerase chain reaction (qRT-PCR) validation

To validate the RNA-seq results, qRT-PCR was performed on selected DEGs. Total RNA was extracted from 30 pooled zebrafish larvae per group at 9 dpf using TRIzol reagent, and complementary DNA was synthesized using a PrimeScript RT reagent kit (Takara, Kusatsu, Japan), following the manufacturer’s instructions. qRT-PCR was conducted using TB Green Premix Ex Taq II (Takara) on a Bio-Rad CFX96 real-time PCR system (Bio-Rad, Hercules, CA, United States). The primers used for qRT-PCR are listed in Supplementary Table S2. Relative gene expression levels were calculated using the 2^-ΔΔCt method, with GAPDH as the reference gene.

### 2.12 Statistical analysis

All data are presented as mean ± standard deviation. Statistical analyses were performed using GraphPad Prism 8 software (GraphPad Software, San Diego, CA United States). Differences among groups were analyzed by one-way analysis of variance followed by Tukey’s *post hoc* test. A P-value of <0.05 was considered statistically significant.

## 3 Results

### 3.1 Effects of TSN on growth and development of zebrafish

**FIGURE 2:**
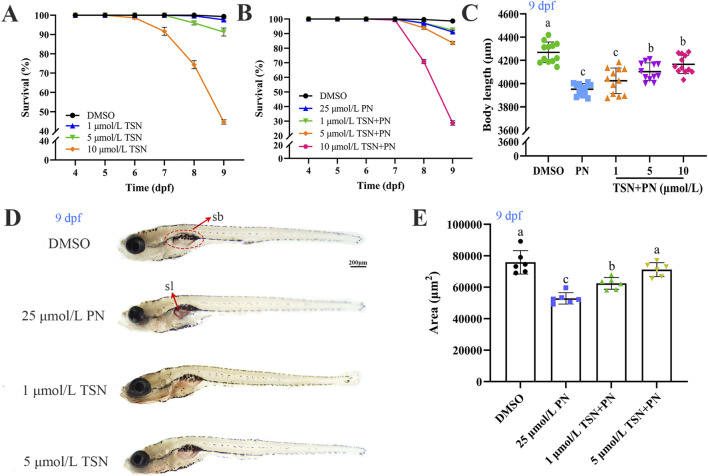
Effects of TSN on the growth and development of juvenile zebrafish. Zebrafish larvae were exposed to various concentrations of TSN and PN for 5 days. **(A)** Survival rate of larvae exposed to different concentrations of TSN. **(B)** Comparison of survival rates among control, PN, and TSN + PN treatment groups. **(C)** Body length measurements across treatment groups (n = 15). **(D)** Lateral view of zebrafish larvae stained with Oil Red O to visualize lipid distribution on the swim bladder (n = 6). **(E)** Quantification of swim bladder area in each group (n = 6). Abbreviations: sb, swim bladder; sl, surface lipid. N = 3 replicate. Error bars represent standard deviation. The significant differences between the different groups were evaluated using one-way ANOVA followed by Tukey’s test. *P* < 0.05 was considered statistically significant. Different letters indicate statistically significant differences.

The effects of TSN on zebrafish larval development were assessed over a 5 days exposure period using a range of concentrations during early developmental stages. To determine the optimal therapeutic concentration, zebrafish larvae were exposed to TSN at 1, 5, and 10 μmol/L, in combination with 25 μmol/L PN. Concentration-dependent effects were observed, and 5 μmol/L TSN was identified as the optimal concentration for subsequent experiments (Figure 2). Compared with the control group, exposure to 25 μmol/L PN significantly increased cumulative mortality at 9 dpf (*P* < 0.05) (Figures 2A,B) and significantly reduced swim bladder size and body length (Figures 2C–E). Additionally, dispersed lipid droplets were observed on the surface of the swim bladder in the PN group (Figure 2D). Interestingly, co-treatment with 5 μmol/L TSN significantly reduced cumulative mortality (*P* < 0.05) (Figures 2A,B) and improved swim bladder size and body length compared with the 25 μmol/L PN group (*P* < 0.05) (Figures 2C–E). These findings suggest that 5 μmol/L TSN mitigates PN-induced developmental toxicity in zebrafish larvae. We observed that 5 μmol/L TSN exhibited no significant lethality or morphological abnormalities compared to the vehicle control, whereas 10 μmol/L induced significant toxicity (Figures 2A,B). Therefore, we then focused on investigating the effect of 5 μml/L TSN in alleviating GIOP in zebrafish larvae.

### 3.2 Effect of TSN on bone mineralization and craniofacial cartilage development

**FIGURE 3:**
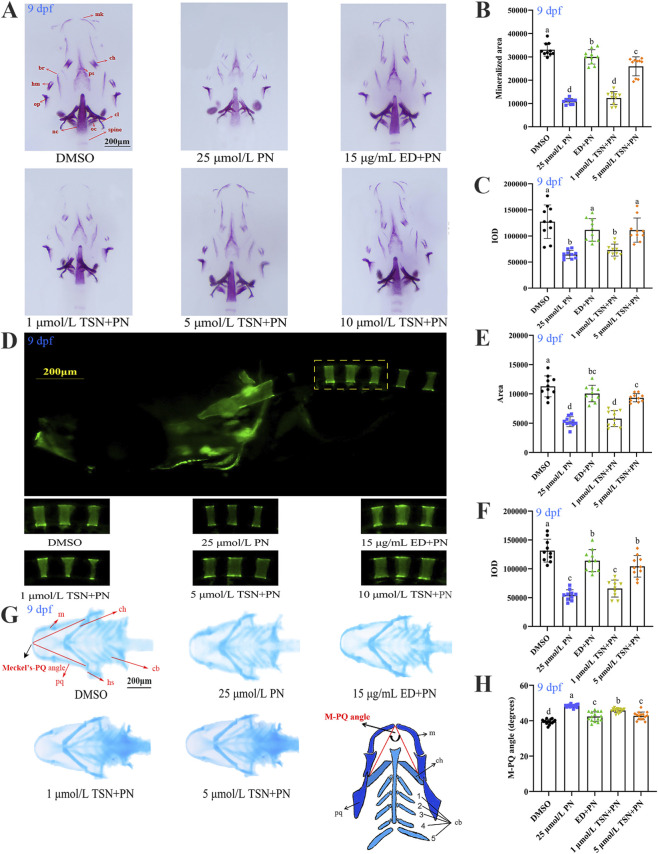
TSN promotes bone regeneration and alleviates cartilage development abnormalities in osteoporotic zebrafish larvae. The six exposure groups included a DMSO control group, 25 μmol/L PN group, 15 μg/mL ED + PN group, and three TSN + PN treatment groups. All treatments were administered for 5 days. **(A)** Ventral view of the skulls of 9 dpf zebrafish larvae stained with alizarin red S. Abbreviations: mk, Meckel’s cartilage; hm, hyoid mandibular arch; op, operculum; cl, cleithrum; ps, parasphenoid bone; oc, occipital bone; nc, notochord; ch, ceratohyal; br, branchial ray. **(B)** Mineralized area measured by quantifying stained skull regions (n = 10). **(C)** IOD of stained skulls assessed by quantitative analysis (n = 10). **(D)** Fluorescence imaging of calcein-stained zebrafish vertebrae using fluorescence microscopy. **(E)** Mineralized area of vertebrae 1–3 in each group determined by quantitative analysis (n = 10). **(F)** IOD of vertebrae 1–3 across treatment groups (n = 10). **(G)** Ventral views of Alcian blue-stained cranial cartilage in zebrafish larvae, showing deep blue cartilage structures. **(H)** Quantitative analysis of the M-PQ angle (highlighted in red), an indicator of craniofacial chondrogenesis (n = 15). Abbreviations: m, Meckel’s cartilage; pq, palatoquadrate; ch, ceratohyal; cb, ceratobranchial. Scale bar = 200 μm. N = 3 replicate. Error bars represent standard deviation. The significant differences between the different groups were evaluated using one-way ANOVA followed by Tukey’s test. *P* < 0.05 was considered statistically significant. Different letters indicate statistically significant differences.

Figures 3A–C illustrates the effect of TSN on the skulls of zebrafish larvae. Compared with the control group, the 25 μmol/L PN group exhibited a significant reduction in both the average staining area and integrated optical density (IOD) (*P* < 0.05). By contrast, the average staining area and IOD in the 15 μg/mL ED group were significantly higher than those in the 25 μmol/L PN group (*P* < 0.05). When TSN was administered at 5 μmol/L, the average staining area and IOD were also significantly greater than in the PN group (*P* < 0.05).

Compared with the control group, treatment with 25 μmol/L PN significantly reduced the fluorescence intensity of the first three vertebrae (V1–V3), indicating bone density loss (Figures 3D–F). A concentration of 15 μg/mL ED reversed this decrease in fluorescence intensity. Treatment with 1 μmol/L TSN did not significantly alter fluorescence intensity compared with the PN group (*P* > 0.05), whereas 5 μmol/L TSN significantly mitigated the PN-induced reduction in vertebral bone density (*P* < 0.05). These findings suggest that 5 μmol/L TSN can protect against PN-induced bone density loss.

The effects of TSN on PN-induced craniofacial cartilage deformities in zebrafish larvae were evaluated by measuring the angle between Meckel’s cartilage and the palatoquadrate (M-PQ angle), a key indicator of chondrogenic abnormalities. Treatment with 25 μmol/L PN significantly increased the M-PQ angle compared with the control group, resulting in a visibly wider anterior skull (Figures 3G,H). By contrast, the M-PQ angle was significantly reduced in larvae treated with 1 and 5 μmol/L TSN, as well as in the 15 μg/mL ED group, compared with the PN group. Notably, the reduction was more pronounced in the 5 μmol/L TSN and 15 μg/mL ED groups, with values approaching those of the control. These results suggest that TSN, particularly at 5 μmol/L, can help mitigate PN-induced craniofacial chondrodysplasia in zebrafish larvae.

### 3.3 Effects of TSN on cardiovascular function and locomotor behavior

**FIGURE 4:**
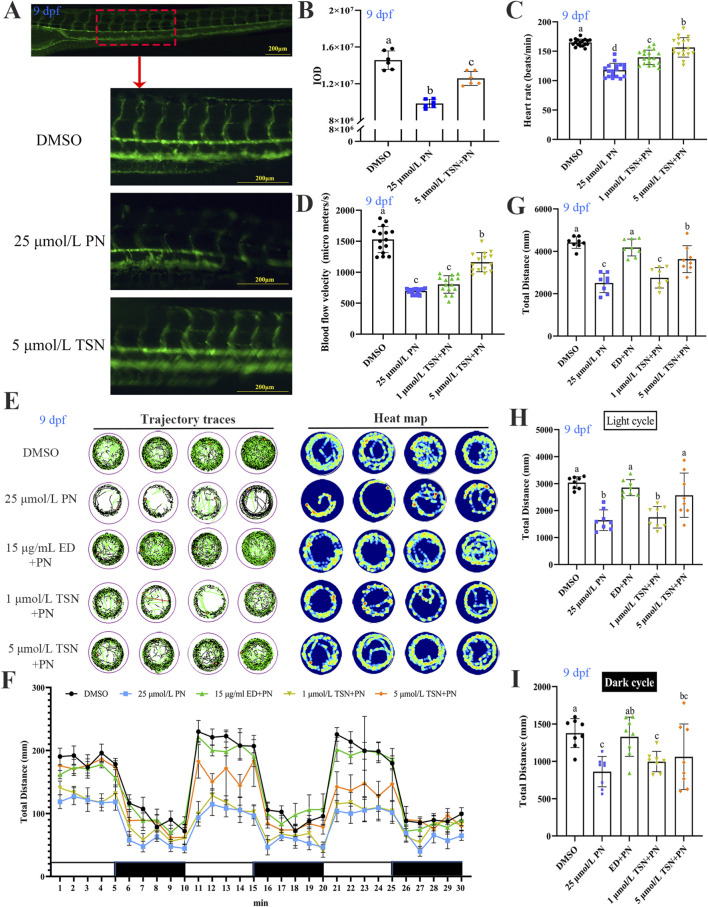
Effects of TSN and PN at different concentrations on cardiovascular function and behavior in juvenile zebrafish. **(A)** Lateral view of vasculature in transgenic *Tg[flk:EGFP]* zebrafish showing internode angiogenesis near the notochord (n = 6). **(B)** IOD of blood vessels in each group, quantified by image analysis (n = 6). **(C)** Heart rate of zebrafish larvae across treatment groups (n = 15). **(D)** Blood flow velocity in zebrafish larvae under different treatments (n = 15). **(E)** Representative locomotor trajectories of 9 dpf zebrafish across five exposure groups over 30 min. **(F)** Mean locomotor activity curves for each group (n = 8). **(G)** Total distance moved by larvae during both dark and light phases within 30 min (n = 8). **(H)** Total distance moved during the dark phase (n = 8). **(I)** Total distance moved during the light phase (n = 8). N = 3 replicate. Error bars represent standard deviation. The significant differences between the different groups were evaluated using one-way ANOVA followed by Tukey’s test. *P* < 0.05 was considered statistically significant. Different letters indicate statistically significant differences.

Compared with the DMSO control group, juvenile zebrafish treated with 25 μmol/L PN showed inhibited formation of new blood vessels (Figures 4A,B). Additionally, PN treatment significantly reduced heart rate, blood flow velocity, and overall activity within 30 min (Figures 4C–F). Locomotor activity during both light and dark cycles also showed a marked reduction in total distance traveled (*P* < 0.05) (Figures 4G–I). In the 1 μmol/L TSN group, total movement distance increased compared with the PN group during both light and dark phases, but the difference was not statistically significant (*P* > 0.05) (Figures 4C,D). However, co-treatment with 5 μmol/L TSN significantly improved heart rate, blood flow velocity, and vascular development compared to the 25 μmol/L PN group (*P* < 0.05) (Figures 4B–D). Furthermore, the total travel distance in the 5 μmol/L TSN group was significantly higher than in the PN group (*P* < 0.05) (Figures 4E–I). These findings suggest that increasing the concentration of TSN can enhance cardiovascular function and locomotor ability in zebrafish larvae.

### 3.4 Effects of TSN on oxidative stress

**FIGURE 5:**
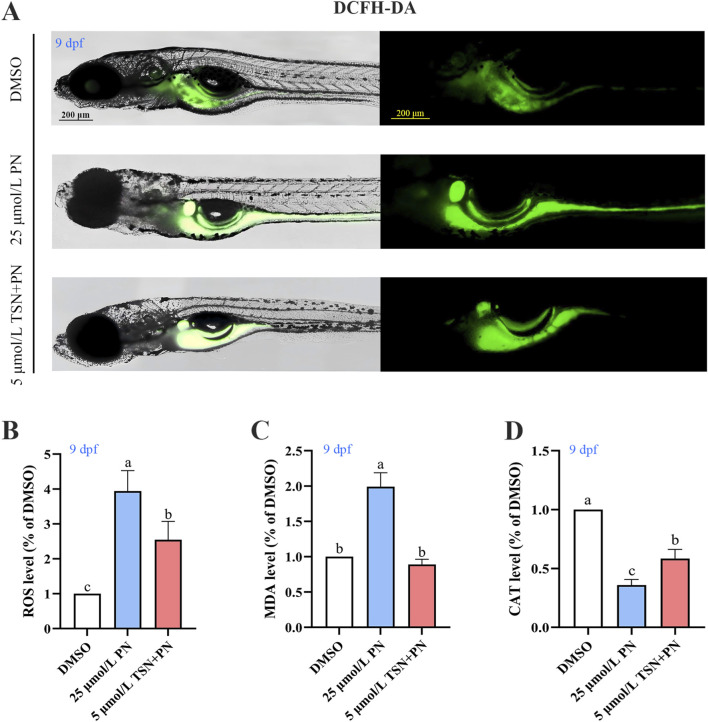
TSN can partially alleviate the oxidative stress induced by PN in zebrafish larvae. **(A)** Representative images of ROS fluorescence. **(B)** ROS levels of zebrafish assessed by quantitative analysis (n = 3). **(C)** CAT activity (n = 3). **(D)** The content of MDA (n = 3). Scale bar = 200 μm. Error bars represent standard deviation. Different letters indicate statistically significant differences (*P* < 0.05) among groups. N = 3 replicate. Error bars represent standard deviation. The significant differences between the different groups were evaluated using one-way ANOVA followed by Tukey’s test. *P* < 0.05 was considered statistically significant. Different letters indicate statistically significant differences.

Compared with the DMSO control group, juvenile zebrafish treated with 25 μmol/L PN showed significantly higher ROS accumulation (*P* < 0.05) (Figures 5A,B). Additionally, PN treatment significantly increased the content of MDA and reduced the CAT activity in the zebrafish larvae (*P* < 0.05) (Figures 5C,D). However, co-treatment with 5 μmol/L TSN significantly reduced ROS level and the content of MDA compared to the 25 μmol/L PN group (*P* < 0.05) (Figures 5A–C). Furthermore, the CAT activity in the 5 μmol/L TSN group was significantly higher than in the PN group (*P* < 0.05) (Figure 5D). These findings suggest that TSN can attenuate the PN-induced increase in oxidative stress in zebrafish larvae.

### 3.5 Whole transcriptome sequencing analysis

#### 3.5.1 Transcriptome assembly and analysis

**FIGURE 6:**
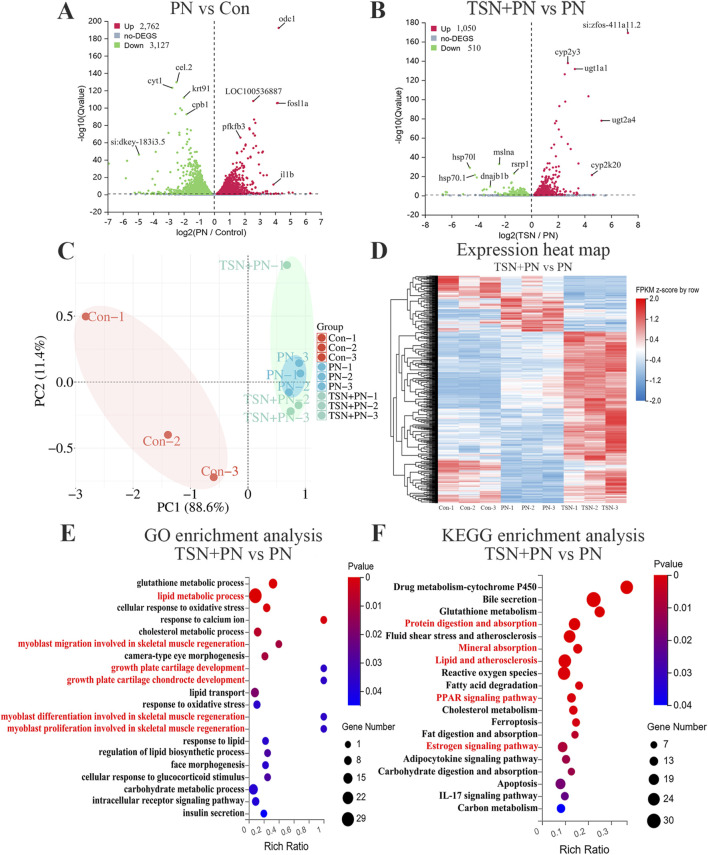
DEGs among the control group, 25 μmol/L PN group, and 5 μmol/L TSN + PN group in RNA-seq analysis. **(A,B)** Volcano plots of DEGs. Red dots represent significantly upregulated genes; green dots represent significantly downregulated genes. **(C)** PCA of transcriptomic profiles in the control, PN, and TSN + PN groups. **(D)** Heatmap showing hierarchical clustering of significantly differentially expressed genes among the three groups. **(E)** GO enrichment analysis of upregulated and downregulated DEGs. **(F)** KEGG pathway enrichment analysis of upregulated and downregulated DEGs.

RNA-seq data revealed substantial gene expression changes among the control group, 25 μmol/L PN group, and 5 μmol/L TSN + PN group. Using DEseq2 algorithms, a total of 5,889 DEGs were identified between the PN and control groups—2,762 upregulated and 3,127 downregulated—among 32,324 genes detected (Figures 6A,B). In comparison, 1,560 DEGs were found between the TSN and PN groups, including 1,050 upregulated and 510 downregulated genes. PCA demonstrated clear separation among the control, PN, and TSN groups, indicating distinct transcriptional profiles (Figure 6C). Hierarchical clustering of DEGs is shown in the accompanying heat map (Figure 6D). To better understand the biological implications of these DEGs, GO classification and functional enrichment analyses were performed. DEGs between PN and Con groups were significantly enriched in lipid metabolism and skeletal system development (Supplementary Figure S1). Furthermore, DEGs between the TSN and PN groups were significantly enriched in processes such as lipid metabolism and myoblast migration involved in skeletal muscle regeneration. KEGG pathway analysis further highlighted functional pathways enriched in the TSN group compared with the PN group. Notably, DEGs were enriched in pathways related to protein digestion and absorption, mineral absorption, lipids and atherosclerosis, the PPAR signaling pathway, and the estrogen signaling pathway (Figures 6E,F).

### 3.6 Functional annotation of DEGseq

**FIGURE 7:**
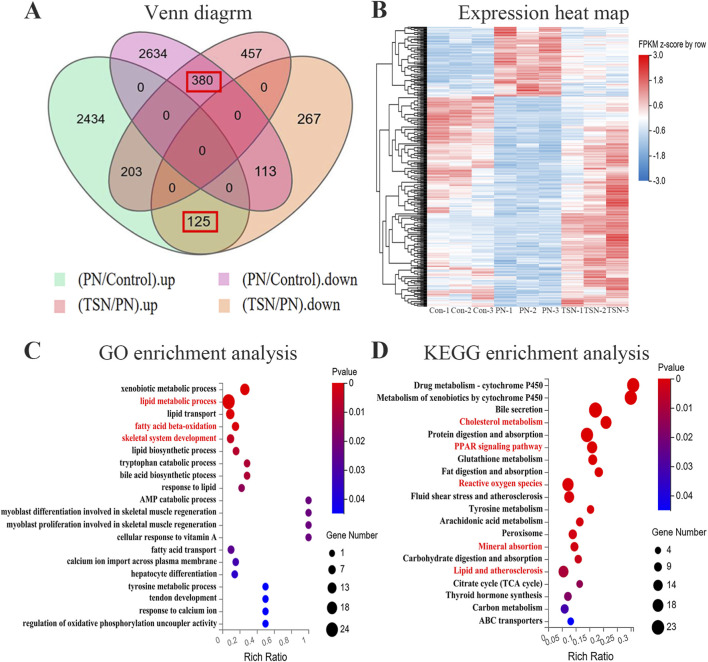
Profile of TSN-induced changes in gene expression. **(A)** Venn diagram showing 505 DEGs whose expression was reversed by TSN treatment. **(B)** Heatmap displaying hierarchical clustering of the 505 reversed DEGs. **(C)** GO enrichment analysis scatterplot of the reversed genes. **(D)** KEGG enrichment analysis scatterplot of the reversed genes.

A Venn diagram revealed 505 “reversed” genes—those whose expression was altered by PN and subsequently reversed by TSN treatment—including 380 upregulated and 125 downregulated genes when comparing the 5 μmol/L TSN + PN group with the 25 μmol/L PN and control groups (Figure 7A). Hierarchical clustering of these genes is shown in the heat map (Figure 7B). To further characterize the regulatory effects of TSN on gene expression in juvenile zebrafish and assess the biological significance of these reversed genes, GO and KEGG enrichment analyses were performed. Based on the GO classification analysis results, the majority of genes associated with cellular components were categorized into “cell” and “cell part”, while genes involved in molecular functions primarily participated in “binding” and “catalytic activity” processes. For biological processes, the dominant categories included “cellular process” (292 DEGs), “metabolic process” (228 DEGs) and “biological regulation” (168 DEGs) (Supplementary Figure S2A). Then, conduct in-depth research on the biological processes aspect of the GO analysis. GO analysis indicated that the reversed genes were primarily involved in skeletal system development, lipid metabolism, and fatty acid β-oxidation (Figure 7C). KEGG pathway analysis revealed enrichment in pathways related to PPAR signaling pathway, lipid atherosclerosis, ROS and cholesterol metabolism (Figure 7D). Notably, the reversed downregulated genes exhibited significant enrichment in pathways related to osteoclast differentiation (Supplementary Figure S2E).

### 3.7 Overview of reversed gene associations

**FIGURE 8:**
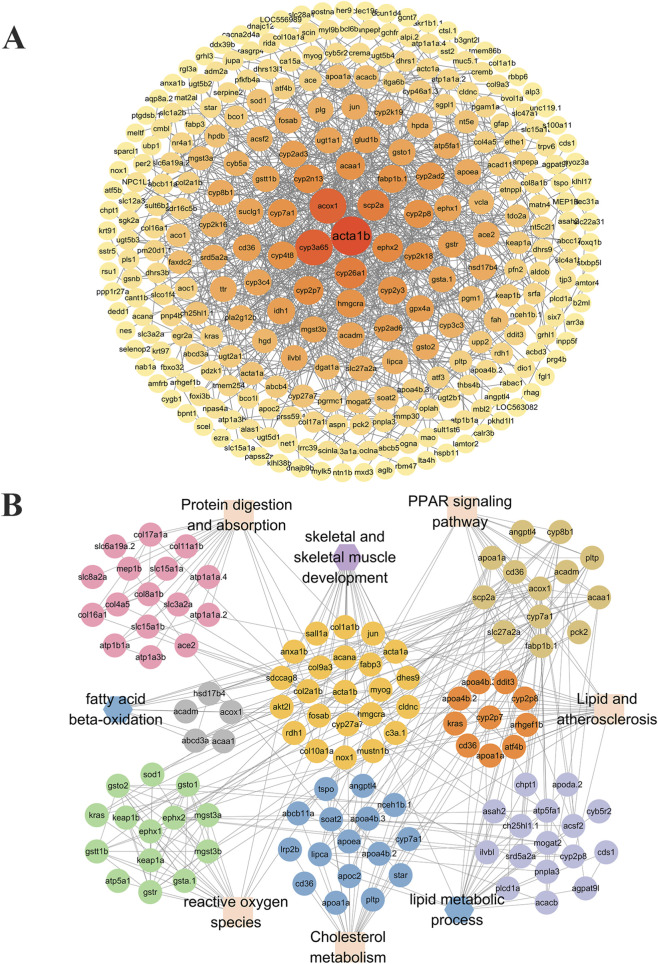
Relationships among reversed genes and TSN target interactions. **(A)** PPIN of the 505 reversed genes. **(B)** PPIN of differentially expressed genes enriched in signaling pathways related to skeletal development.

The PPIN map illustrates the relationships among the reversed genes, highlighting strong correlations for acta1b, acox1, and cyp3a65 (Figure 8A). The PPIN also showed that among the 505 reversed genes, the PPAR and ROS signaling pathways were most closely associated with skeletal and skeletal muscle development (Figure 8B).

### 3.8 Verification of transcriptomic sequencing using qRT-PCR

**FIGURE 9:**
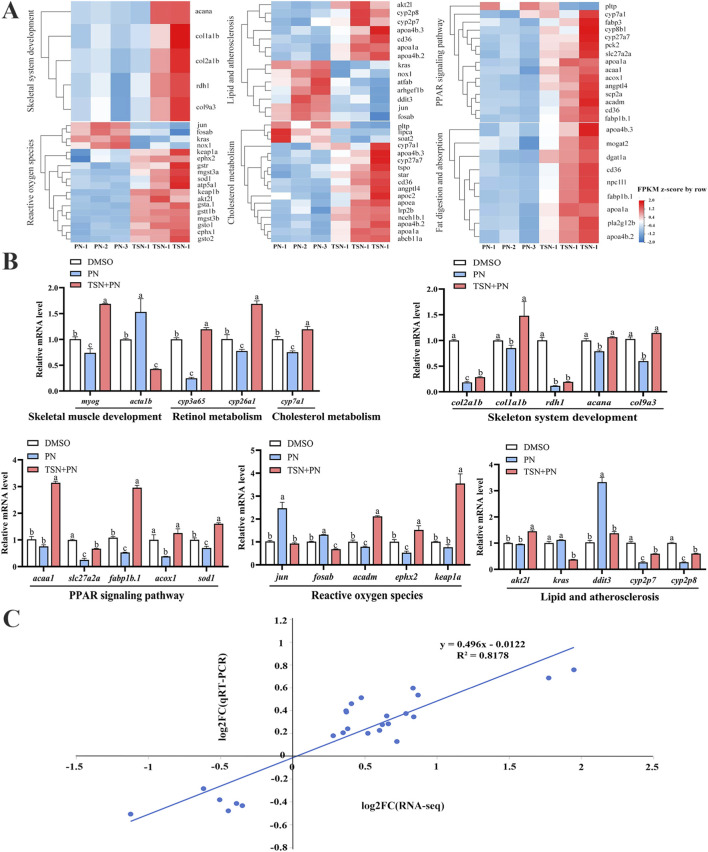
Comprehensive analysis of key signaling pathways in skeletal development: expression profiles and transcriptomic validation. **(A)** Heatmap of gene expression in key signaling pathways related to skeletal development based on RNA-seq data. **(B)** qRT-PCR validation of gene expression across treatment groups. Twenty-five DEGs identified by RNA-seq were selected for validation and compared with RNA-seq results. **(C)** Correlation analysis between RNA-seq and qRT-PCR results. Dotted lines represent RNA-seq data; dots represent qRT-PCR data. *R* ^*2*^ indicates the correlation coefficient, with higher values reflecting stronger agreement. The x-axis shows log_2_ fold changes from RNA-seq, and the y-axis shows log_2_ fold changes from qRT-PCR.

The heatmap of mRNA expression for key genes involved in skeletal development from the RNA-seq data showed that these genes were reversed following TSN treatment (Figure 9A). To validate the transcriptomic results, 25 signature genes related to skeletal development were analyzed by qRT-PCR. Although minor differences in expression levels were observed, the overall expression patterns were largely consistent with those identified by high-throughput sequencing (Figure 9B). A significant positive correlation was observed between the fold changes in expression of 25 selected DEGs from RNA-seq and qRT-PCR, with a correlation coefficient (*R* ^2^) of 0.8178, as determined by linear regression analysis (Figure 9C).

## 4 Discussion

The present study investigated the protective efficacy of TSN against GIOP using a PN-induced zebrafish model. The experimental protocol involved exposure to 25 μmol/L PN, a concentration previously validated for inducing osteoporosis in zebrafish larvae (Jiang et al., 2024). Osteoporosis is a serious metabolic bone disorder characterized by reduced bone mass and an increased risk of fractures (Formosa et al., 2024), representing a significant public health concern, particularly among aging populations (Shevroja et al., 2023). Although PN is widely used in the treatment of various diseases (Son et al., 2023), its prolonged use frequently results in secondary osteoporosis, negatively impacting patient outcomes and quality of life (Tanaka et al., 2024). Zebrafish were chosen as the model organism because of their conserved skeletal development and suitability for high-throughput analysis. To investigate the mechanisms by which TSN mitigates GIOP, we employed an integrative approach combining morphological assessment, transcriptomic sequencing, and molecular biology techniques. This comprehensive strategy enabled us to evaluate the therapeutic potential of TSN and uncover its underlying molecular mechanisms, offering a novel framework for studying traditional Chinese herbal medicines in the context of GIOP. Our findings demonstrate that 5 μmol/L TSN confers strong protection against PN-induced systemic toxicity, particularly in reducing skeletal damage. Additionally, bone health is closely related to vascular health. This relationship involves the vascular-skeletal microenvironment. The vascular system supplies oxygen and nutrients to bone tissue. It also transports hormones that regulate bone metabolism. These hormones directly or indirectly influence the differentiation and activity of osteoblasts and osteoclasts (Kusumbe et al., 2014). Physical activity serves as a functional indicator of the musculoskeletal system. It reflects both bone integrity and muscle function. In GIOP, both bone integrity and muscle function are often affected simultaneously.

Transcriptomics provides a powerful means of dynamically monitoring comprehensive molecular responses during the early stages of traditional Chinese herbal medicine interventions. This approach not only enables precise elucidation of the spatiotemporal dynamics of multi-target mechanisms underlying physiological adaptation, but it also helps establish a cross-omics evidence chain—linking molecular interactions to pathological improvements through differential gene module–phenotype correlation analysis. This systems biology framework facilitates exploration of the synergistic mechanisms by which traditional herbal medicine mitigates GIOP. In our study, GO enrichment analysis of reversed genes revealed that those exhibiting inverse expression patterns following TSN treatment were predominantly enriched in three functionally interconnected biological processes, including skeletal system development (Figure 7C). These findings support the dual function of TSN in both mitigating GIOP and promoting osteogenesis. Notably, the progression of skeletal development was associated with a marked upregulation of extracellular matrix components, collagen family proteins, and regulators of cartilage formation-elements typically suppressed during GC exposure. This observation aligns with previous literature highlighting TSN’s osteogenic effects, mediated through the modulation of canonical bone formation pathways such as Wnt/β-catenin signaling, BMP-SMAD activation, and the PI3K/AKT/mTOR cascade (Kim and Kim, 2010; Zhou et al., 2020; Zeng and Bao, 2021).

Our GO enrichment analysis confirmed that the reversed genes were significantly enriched in lipid metabolic processes and fatty acid β-oxidation. This suggests that lipid metabolism may play a key role in mediating the therapeutic effects of TSN in alleviating GIOP, providing molecular evidence for the regulatory role of lipid catabolism in bone remodeling. Fatty acid β-oxidation, which occurs in both mitochondria and peroxisomes, is a highly efficient energy-generating process that critically supports the ATP-demanding functions of osteoblast differentiation, including chromatin remodeling and extracellular matrix mineralization (Shen et al., 2022). Mechanistically, each cycle of fatty acid β-oxidation generates two high-energy molecules that are subsequently oxidized via the mitochondrial electron transport chain, driving ATP synthesis through oxidative phosphorylation and alleviating the energy deficit induced by GCs (Enkler et al., 2023). Our study demonstrated that TSN significantly enhances the fatty acid β-oxidation process, consistent with previous research findings (Gao et al., 2021). Furthermore, fatty acid β-oxidation helps mitigate GC-induced lipid accumulation by accelerating the breakdown of free fatty acids within bone marrow mesenchymal stem cells (Tabe et al., 2017), thereby reducing the abnormal buildup of lipid droplets (Gao and Chen, 2022). Ultimately, this process upregulates osteogenic markers in bone marrow mesenchymal stem cells while downregulating adipogenic markers, restoring the balance between osteogenic and adipogenic differentiation and enhancing bone formation capacity (Wang et al., 2024; Li et al., 2025). In agreement with established findings, the TSN-induced promotion of fatty acid oxidation not only mitigates lipotoxicity but also restores energy metabolism balance. This, in turn, synergistically counteracts the GC-induced suppression of osteogenic differentiation (Yuan et al., 2022; Wang et al., 2024).

Experimental evidence demonstrating the coordinated enhancement of these three interrelated biological processes confirms that TSN functions as a multi-target therapeutic agent. It effectively addresses two primary pathological outcomes induced by GCs: impaired bone formation and lipid accumulation. This multifaceted approach offers a more comprehensive strategy for managing GIOP compared with conventional antiresorptive therapies, which primarily target osteoclast inhibition. TSN directly rectifies deficiencies in osteoblastic bone formation and lipid metabolic reprogramming—both critical for preserving skeletal integrity and functional outcomes (Tamura et al., 2004; Loomba et al., 2021). In contrast to traditional GIOP interventions, TSN’s broader mechanism of action addresses the root causes of skeletal deterioration.

This study showed that TSN modulates key pathways involved in cholesterol metabolism, PPAR signaling, and lipid atherosclerosis. TSN effectively restored the cholesterol metabolism pathway, thereby mitigating the adverse effects of hypercholesterolemia on trabecular bone. This finding is consistent with existing literature, which establishes a strong correlation between elevated cholesterol levels and increased fracture risk (Dai et al., 2021; Liu X. et al., 2023). Additionally, TSN rebalanced PPAR signaling, targeting a well-established molecular switch that governs mesenchymal stem cell fate (Li et al., 2023). This regulatory effect counteracted the pro-adipogenic influence of PN, supporting prior evidence that restoration of the PPAR signaling pathway can alleviate GC-induced skeletal integrity compromise (Suo et al., 2022). This mechanism counteracts glucocorticoid-induced adverse effects in a multi-faceted manner, primarily by reversing the PPARα- and PPARγ-mediated suppression of bone formation and potentiation of adipogenesis, while potentially promoting osteogenesis and fatty acid oxidation through PPARβ/δ activation (Chougule et al., 2023; Liu Y. et al., 2023; Zhang et al., 2023; Pi et al., 2024). Concurrently, TSN intervention corrected dysregulated lipid metabolism and attenuated pathways associated with atherosclerosis, potentially enhancing osteogenic angiogenesis through remodeling of the vascular niche (Ren et al., 2019). The atherosclerotic vascular component plays a critical role in skeletal pathophysiology, with epidemiological studies highlighting a bidirectional relationship between vascular calcification and osteoporotic fractures, mediated by converging mechanisms (Chang et al., 2023). The PPAR signaling pathway intersects functionally with cholesterol metabolism and the regulation of lipid and atherosclerotic processes. TSN effectively restores lipid homeostasis disrupted by GC-induced dyslipidemia—a key contributor to skeletal vascular insufficiency and osteoblast dysfunction (Pi et al., 2024). This synergistic effect enhances skeletal vascular perfusion, supporting bone remodeling and contributing to the maintenance of osseous homeostasis (Kim et al., 2021).

The findings from this study suggest that TSN attenuates the ROS pathway, potentially mitigating oxidative stress–induced osteoblast apoptosis and excessive osteoclast activity—mechanisms that have been thoroughly validated in GC-induced bone deterioration (Chen et al., 2020; Li et al., 2025). Unlike ED, which primarily functions by directly inhibiting osteoclast activity, TSN employs a multi-targeted approach that preserves the therapeutic efficacy of GCs while addressing the underlying metabolic causes of bone loss. Importantly, these pathways operate as an integrated network rather than in isolation; the ROS signaling pathway interacts dynamically with the PPAR signaling pathway, cholesterol metabolism, and lipid atherosclerosis pathways. Activation of PPARγ enhances the expression of antioxidant enzymes, thereby directly neutralizing ROS and facilitating the repair of oxidative damage (Miao et al., 2021). Simultaneously, optimized cholesterol metabolism reduces bone marrow adiposity and mitigates lipid atherosclerotic responses by decreasing circulating low-density lipoprotein levels and inhibiting the deposition of oxidized low-density lipoprotein in endothelial cells and macrophages (Haghikia et al., 2022), further contributing to ROS neutralization and oxidative damage repair (Tan et al., 2019). As a result, this process alleviates lipid atherosclerosis and improves skeletal blood supply, thereby reducing local hypoxia-induced ROS surges (Jia et al., 2016; Chen et al., 2022). Collectively, these mechanisms disrupt the cascade of ROS, inflammation, and lipid disorders, preserving osteoblast function, inhibiting osteoclast hyperactivation, and maintaining vascular bone homeostasis in the context of GIOP. This interconnected mechanism aligns with recent studies identifying the interplay between PPAR signaling, lipid metabolism, and ROS as a promising therapeutic target for GIOP (Jiang et al., 2016; Xia et al., 2024).

This study provides compelling evidence for the therapeutic potential of TSN in a zebrafish model of GIOP. However, further research is needed to substantiate these findings. Zebrafish larvae undergo rapid bone development and mineralization. Their physiological processes are predominantly driven by bone formation. This differs fundamentally from the imbalance in bone remodeling observed in adult glucocorticoid-induced osteoporosis. In adult osteoporosis, the condition is characterized by suppressed bone formation and enhanced bone resorption. Thus, although the present model is appropriate for analyzing the direct osteogenic effects of the drug, it cannot recapitulate the enhanced osteoclast-mediated bone resorption or its complex coupling with bone formation typical of adult osteoporosis. Future studies should further verify the preventive and therapeutic effects of tanshinone II A in mature animal models. Suitable models may include ovariectomized rats, mammals subjected to long-term glucocorticoid treatment, or adult zebrafish. Such models would allow a more systematic evaluation of the compound’s role in regulating the bidirectional balance of bone metabolism. They would also provide experimental evidence more closely aligned with actual pathophysiology to support clinical translation. Additionally, a more detailed examination of specific molecular interactions within the identified pathways—and how these are modulated by TSN—is essential for a comprehensive understanding of its mechanism of action. Our conclusions are primarily drawn from transcriptional regulation data. Future studies should incorporate protein-level validation. This approach will provide stronger support and fully clarify the molecular mechanism through which TSN mitigates osteoporosis. Exploring various routes of administration and formulations of TSN may also be critical for advancing its clinical application. Overall, this research lays a strong foundation for the continued investigation of TSN as a promising candidate for the prevention and treatment of GIOP.

## 5 Conclusion

This study confirmed the substantial osteoprotective effects of TSN in a PN-induced larval zebrafish model of GIOP. Our findings demonstrate that TSN holds significant therapeutic potential as a natural compound for mitigating osteoporosis progression and reducing cartilage abnormalities. The underlying mechanisms appear to involve modulation of key mediators within skeletal development pathways. Additionally, TSN was found to ameliorate GIOP by influencing critical biological processes, including PPAR signaling, lipid metabolism, and ROS regulation. These results underscore the promise of TSN as a novel therapeutic agent for osteoporosis, warranting further mechanistic studies and preclinical validation to confirm its efficacy and mechanism-based action.

## Data Availability

The original contributions presented in the study are included in the article/Supplementary Material, further inquiries can be directed to the corresponding authors.

## References

[B1] Chang G. R. Cheng W. Y. Fan H. C. Chen H. L. Lan Y. W. Chen M. S. (2023). Kefir peptides attenuate atherosclerotic vascular calcification and osteoporosis in atherogenic diet-fed ApoE (-/-) knockout mice. Front. Cell. Dev. Biol. 11, 1158812. 10.3389/fcell.2023.1158812 37091976 PMC10117689

[B2] Chapurlat R. Bui M. Sornay-Rendu E. Zebaze R. Delmas P. D. Liew D. (2020). Deterioration of cortical and trabecular microstructure identifies women with osteopenia or normal bone mineral density at imminent and long-term risk for fragility fracture: a prospective study. J. Bone Min. Res. 35 (5), 833–844. 10.1002/jbmr.3924 31821619 PMC9328422

[B3] Chen W. Yuan C. Lu Y. Zhu Q. Ma X. Xiao W. (2020). Tanshinone IIA protects against acute pancreatitis in mice by inhibiting oxidative stress *via* the Nrf2/ROS pathway. Oxid. Med. Cell. Longev. 2020, 5390482. 10.1155/2020/5390482 32322336 PMC7168729

[B4] Chen X. Han D. Liu T. Huang C. Hu Z. Tan X. (2022). Asiatic acid improves high-fat-diet-induced osteoporosis in mice *via* regulating SIRT1/FOXO1 signaling and inhibiting oxidative stress. Histol. Histopathol. 37 (8), 769–777. 10.14670/HH-18-446 35229281

[B5] Chen M. Fu W. Xu H. Liu C. J. (2023). Pathogenic mechanisms of glucocorticoid-induced osteoporosis. Cytokine Growth Factor Rev. 70, 54–66. 10.1016/j.cytogfr.2023.03.002 36906448 PMC10518688

[B6] Chiodini I. Merlotti D. Falchetti A. Gennari L. (2020). Treatment options for glucocorticoid-induced osteoporosis. Expert Opin. Pharmacother. 21 (6), 721–732. 10.1080/14656566.2020.1721467 32004105

[B7] Chougule A. Baroi S. Czernik P. J. Crowe E. Chang M. R. Griffin P. R. (2023). Osteocytes contribute *via* nuclear receptor PPAR-alpha to maintenance of bone and systemic energy metabolism. Front. Endocrinol. (Lausanne) 14, 1145467. 10.3389/fendo.2023.1145467 37181042 PMC10173151

[B8] Dai B. Li X. Xu J. Zhu Y. Huang L. Tong W. (2021). Synergistic effects of magnesium ions and simvastatin on attenuation of high-fat diet-induced bone loss. Bioact. Mater. 6 (8), 2511–2522. 10.1016/j.bioactmat.2021.01.027 33665494 PMC7889436

[B9] Enkler L. Szentgyörgyi V. Pennauer M. Prescianotto-Baschong C. Riezman I. Wiesyk A. (2023). Arf1 coordinates fatty acid metabolism and mitochondrial homeostasis. Nat. Cell. Biol. 25 (8), 1157–1172. 10.1038/s41556-023-01180-2 37400497 PMC10415182

[B10] Fan X. Zhang Y. Li X. Ding J. Huang J. Lian K. (2025). Unraveling ginsenoside Rg1's osteoprotective pathways in zebrafish models of glucocorticoid induced osteoporosis *via* transcriptomics. Sci. Rep. 15, 30519. 10.1038/s41598-025-15284-2 40835685 PMC12368200

[B11] Formosa M. M. Christou M. A. Mäkitie O. (2024). Bone fragility and osteoporosis in children and young adults. J. Endocrinol. Invest. 47 (2), 285–298. 10.1007/s40618-023-02179-0 37668887 PMC10859323

[B12] Ganesan K. Goyal A. Roane D. (2024). StatPearls. Treasure Island (FL).

[B13] Gao Z. Chen X. (2022). Fatty acid β-Oxidation in kidney diseases: perspectives on pathophysiological mechanisms and therapeutic opportunities. Front. Pharmacol. 13, 805281. 10.3389/fphar.2022.805281 35517820 PMC9065343

[B14] Gao W. Y. Chen P. Y. Hsu H. J. Lin C. Y. Wu M. J. Yen J. H. (2021). Tanshinone IIA downregulates lipogenic gene expression and attenuates lipid accumulation through the modulation of LXRα/SREBP1 pathway in HepG2 cells. Biomedicines 9 (3), 326. 10.3390/biomedicines9030326 33806955 PMC8004631

[B15] Guo Y. Li Y. Xue L. Severino R. P. Gao S. Niu J. (2014). Salvia miltiorrhiza: an ancient Chinese herbal medicine as a source for anti-osteoporotic drugs. J. Ethnopharmacol. 155 (3), 1401–1416. 10.1016/j.jep.2014.07.058 25109459

[B16] Haghikia A. Zimmermann F. Schumann P. Jasina A. Roessler J. Schmidt D. (2022). Propionate attenuates atherosclerosis by immune-dependent regulation of intestinal cholesterol metabolism. Eur. Heart J. 43 (6), 518–533. 10.1093/eurheartj/ehab644 34597388 PMC9097250

[B17] Hildebrand G. K. Patel P. Kasi A. (2024). StatPearls. Treasure Island (FL).

[B18] Howe K. Clark M. D. Torroja C. F. Torrance J. Berthelot C. Muffato M. (2013). The zebrafish reference genome sequence and its relationship to the human genome. Nature 496 (7446), 498–503. 10.1038/nature12111 23594743 PMC3703927

[B19] Jia L. Q. Zhang N. Xu Y. Chen W. n. Zhu M. l. Song N. (2016). Tanshinone IIA affects the HDL subfractions distribution not serum lipid levels: involving in intake and efflux of cholesterol. Arch. Biochem. Biophys. 592, 50–59. 10.1016/j.abb.2016.01.001 26820219

[B20] Jiang Y. Gou H. Wang S. Zhu J. Tian S. Yu L. (2016). Effect of pulsed electromagnetic field on bone formation and lipid metabolism of glucocorticoid-induced osteoporosis rats through canonical wnt signaling pathway. Evid. Based Complement. Altern. Med. 2016, 4927035. 10.1155/2016/4927035 26941827 PMC4749801

[B21] Jiang Z. Deng L. Li M. Alonge E. Wang Y. Wang Y. (2024). Ginsenoside Rg1 modulates PI3K/AKT pathway for enhanced osteogenesis *via* GPER. Phytomedicine 124, 155284. 10.1016/j.phymed.2023.155284 38176267

[B22] Kim H. J. Kim S. H. (2010). Tanshinone IIA enhances BMP-2-stimulated commitment of C2C12 cells into osteoblasts *via* p38 activation. Amino Acids 39 (5), 1217–1226. 10.1007/s00726-010-0557-8 20300786

[B23] Kim H. Oh B. Park-Min K. H. (2021). Regulation of osteoclast differentiation and activity by lipid metabolism. Cells 10 (1), 89. 10.3390/cells10010089 33430327 PMC7825801

[B24] Kum K. Y. Kirchhof R. Luick R. Heinrich M. (2021). Danshen (Salvia miltiorrhiza) on the global market: what are the implications for products' quality. Front. Pharmacol. 12, 621169. 10.3389/fphar.2021.621169 33981218 PMC8107819

[B25] Kusumbe A. P. Ramasamy S. K. Adams R. H. (2014). Coupling of angiogenesis and osteogenesis by a specific vessel subtype in bone. Nature 507, 323–328. 10.1038/nature13145 24646994 PMC4943525

[B26] Lane N. E. (2019). Glucocorticoid-induced osteoporosis: new insights into the pathophysiology and treatments. Curr. Osteoporos. Rep. 17 (1), 1–7. 10.1007/s11914-019-00498-x 30685820 PMC6839409

[B27] Lewiecki E. M. (2010). Bisphosphonates for the treatment of osteoporosis: insights for clinicians. Ther. Adv. Chronic Dis. 1 (3), 115–128. 10.1177/2040622310374783 23251734 PMC3513863

[B28] Li F. Q. Zeng D. K. Jia C. L. Zhou P. Yin L. Zhang B. (2015). The effects of sodium tanshinone IIa sulfonate pretreatment on high glucose-induced expression of fractalkine and apoptosis in human umbilical vein endothelial cells. Int. J. Clin. Exp. Med. 8 (4), 5279–5286. 26131102 PMC4483885

[B29] Li S. Yang K. Cao W. Guo R. Liu Z. Zhang J. (2023). Tanshinone IIA enhances the therapeutic efficacy of mesenchymal stem cells derived exosomes in myocardial ischemia/reperfusion injury *via* up-regulating miR-223-5p. J. Control Release 358, 13–26. 10.1016/j.jconrel.2023.04.014 37086952

[B30] Li X. Yang X. Liu Z. Liu H. Lv H. Li X. (2025). Tanshinone IIA reverses osteogenic differentiation of bone marrow mesenchymal stromal cells impaired by glucocorticoids *via* the ERK1/2-CREB signaling pathway. Chem. Biol. Drug Des. 105 (3), e70069. 10.1111/cbdd.70069 40047141

[B31] Liu X. Gu Y. Kumar S. Amin S. Guo Q. Wang J. (2023). Oxylipin-PPARγ-initiated adipocyte senescence propagates secondary senescence in the bone marrow. Cell. Metab. 35, 667–684.e6. 10.1016/j.cmet.2023.03.005 37019080 PMC10127143

[B32] Liu Y. Jia Z. Ma L. Wang D. (2023). Pyrophosphorylated-cholesterol-modified bone-targeting liposome formulation procedure. Methods Mol. Biol. 2622, 207–220. 10.1007/978-1-0716-2954-3_18 36781763

[B33] Loomba R. Friedman S. L. Shulman G. I. (2021). Mechanisms and disease consequences of nonalcoholic fatty liver disease. Cell. 184 (10), 2537–2564. 10.1016/j.cell.2021.04.015 33989548 PMC12168897

[B34] Lu J. Hu D. Zhang Y. Ma C. Shen L. Shuai B. (2023). Current comprehensive understanding of denosumab (the RANKL neutralizing antibody) in the treatment of bone metastasis of malignant tumors, including pharmacological mechanism and clinical trials. Front. Oncol. 13, 1133828. 10.3389/fonc.2023.1133828 36860316 PMC9969102

[B35] Ma X. Zhang L. Gao F. Jia W. Li C. (2023). Salvia miltiorrhiza and tanshinone IIA reduce endothelial inflammation and atherosclerotic plaque formation through inhibiting COX-2. Biomed. Pharmacother. 167, 115501. 10.1016/j.biopha.2023.115501 37713995

[B36] Miao Y. Zheng Y. Geng Y. Yang L. Cao N. Dai Y. (2021). The role of GLS1-mediated glutaminolysis/2-HG/H3K4me3 and GSH/ROS signals in Th17 responses counteracted by PPARγ agonists. Theranostics 11 (9), 4531–4548. 10.7150/thno.54803 33754076 PMC7977454

[B37] Peng C. H. Lin W. Y. Yeh K. T. Chen I. H. Wu W. T. Lin M. D. (2021). The molecular etiology and treatment of glucocorticoid-induced osteoporosis. Tzu Chi Med. J. 33 (3), 212–223. 10.4103/tcmj.tcmj_233_20 34386357 PMC8323641

[B38] Pi D. Liang Z. Pan J. Zhen J. Zheng C. Fan W. (2024). Tanshinone IIA inhibits the endoplasmic reticulum stress-induced unfolded protein response by activating the PPARα/FGF21 axis to ameliorate nonalcoholic steatohepatitis. Antioxidants Basel, Switz. 13, 1026. 10.3390/antiox13091026 39334685 PMC11428933

[B39] Queiroz Júnior J. Cartaxo M. Paz S. T. Fdcâm T. Lemos A. Maia C. S. (2022). Histomorphometry of bone microarchitecture in rats treated with vitamin D and bisphosphonate in the management of osteoporosis. Rev. Bras. Ortop. (Sao Paulo) 57 (2), 267–272. 10.1055/s-0041-1741023 35652013 PMC9142217

[B40] Ren J. Fu L. Nile S. H. Zhang J. Kai G. (2019). Salvia miltiorrhiza in treating cardiovascular diseases: a review on its pharmacological and clinical applications. Front. Pharmacol. 10, 753. 10.3389/fphar.2019.00753 31338034 PMC6626924

[B41] Shen L. Hu G. Karner C. M. (2022). Bioenergetic metabolism in osteoblast differentiation. Curr. Osteoporos. Rep. 20 (1), 53–64. 10.1007/s11914-022-00721-2 35112289 PMC9245007

[B42] Shevroja E. Reginster J. Y. Lamy O. Al-Daghri N. Chandran M. Demoux-Baiada A. L. (2023). Update on the clinical use of trabecular bone score (TBS) in the management of osteoporosis: results of an expert group meeting organized by the European society for clinical and economic aspects of osteoporosis, osteoarthritis and musculoskeletal diseases (ESCEO), and the international osteoporosis foundation (IOF) under the auspices of WHO collaborating center for epidemiology of musculoskeletal health and aging. Osteoporos. Int. 34 (9), 1501–1529. 10.1007/s00198-023-06817-4 37393412 PMC10427549

[B43] Son Y. Kim B. Y. Kim M. Kim J. Kwon R. J. Kim K. (2023). Glucocorticoids impair the 7α-Hydroxycholesterol-Enhanced innate immune response. Immune Netw. 23 (5), e40. 10.4110/in.2023.23.e40 37970232 PMC10643330

[B44] Steinbuch M. Youket T. E. Cohen S. (2004). Oral glucocorticoid use is associated with an increased risk of fracture. Osteoporos. Int. 15 (4), 323–328. 10.1007/s00198-003-1548-3 14762652

[B45] Suo J. Zou S. Wang J. Han Y. Zhang L. Lv C. (2022). The RNA-Binding protein Musashi2 governs osteoblast-adipocyte lineage commitment by suppressing PPARγ signaling. Bone Res. 10 (1), 31. 10.1038/s41413-022-00202-3 35301280 PMC8930990

[B46] Tabe Y. Yamamoto S. Saitoh K. Sekihara K. Monma N. Ikeo K. (2017). Bone marrow adipocytes facilitate fatty acid oxidation activating AMPK and a transcriptional network supporting survival of acute monocytic leukemia cells. Cancer Res. 77 (6), 1453–1464. 10.1158/0008-5472.CAN-16-1645 28108519 PMC5354955

[B47] Tamura Y. Okinaga H. Takami H. (2004). Glucocorticoid-induced osteoporosis. Biomed. Pharmacother. 58 (9), 500–504. 10.1016/j.biopha.2004.08.018 15511606

[B48] Tan Y. L. Ou H. X. Zhang M. Gong D. Zhao Z. W. Chen L. Y. (2019). Tanshinone IIA promotes macrophage cholesterol efflux and attenuates atherosclerosis of apoE-/- mice by Omentin-1/ABCA1 pathway. Curr. Pharm. Biotechnol. 20 (5), 422–432. 10.2174/1389201020666190404125213 30947667

[B49] Tanaka Y. Soen S. Hirata S. Okada Y. Fujiwara S. Tanaka I. (2024). The 2023 guidelines for the management and treatment of glucocorticoid-induced osteoporosis. J. Bone Min. Metab. 42 (2), 143–154. 10.1007/s00774-024-01502-w 38538869 PMC10982086

[B50] Timmermans S. Souffriau J. Libert C. (2019). A general introduction to glucocorticoid biology. Front. Immunol. 10, 1545. 10.3389/fimmu.2019.01545 31333672 PMC6621919

[B51] Wang L. Ma R. Liu C. Liu H. Zhu R. Guo S. (2017). Salvia miltiorrhiza: a potential red light to the development of cardiovascular diseases. Curr. Pharm. Des. 23 (7), 1077–1097. 10.2174/1381612822666161010105242 27748194 PMC5421141

[B52] Wang W. Wu H. Feng S. Huang X. Xu H. Shen X. (2024). Tanshinone IIA promotes osteogenic differentiation potential and suppresses adipogenic differentiation potential of bone marrow mesenchymal stem cells. Mol. Med. Rep. 30 (4), 177. 10.3892/mmr.2024.13301 39129299 PMC11332326

[B53] Weinstein R. S. (2012). Glucocorticoid-induced osteoporosis and osteonecrosis. Endocrinol. Metab. Clin. North Am. 41 (3), 595–611. 10.1016/j.ecl.2012.04.004 22877431 PMC3417039

[B54] Xia B. Dai X. Shi H. Yin J. Xu T. Liu T. (2024). Lycopene promotes osteogenesis and reduces adipogenesis through regulating FoxO1/PPARγ signaling in ovariectomized rats and bone marrow mesenchymal stem cells. Nutrients 16 (10), 1443. 10.3390/nu16101443 38794681 PMC11123960

[B55] Yang H. Zhang H. Tong X. Zhang J. Shen Y. (2019). Recovery of chicken growth plate by TanshinoneIIA through wnt/β-catenin pathway in thiram-induced tibial dyschondroplasia. Ecotoxicol. Environ. Saf. 183, 109575. 10.1016/j.ecoenv.2019.109575 31442808

[B56] Yuan P. Qin H. Y. Wei J. Y. Chen G. Li X. (2022). Proteomics reveals the potential mechanism of tanshinone IIA in promoting the *Ex Vivo* expansion of human bone marrow mesenchymal stem cells. Regen. Ther. 21, 560–573. 10.1016/j.reth.2022.11.004 36475023 PMC9700269

[B57] Zeng J. Bao X. (2021). Tanshinone IIA attenuates high glucose-induced epithelial-to-mesenchymal transition in HK-2 cells through VDR/Wnt/β-catenin signaling pathway. Folia Histochem Cytobiol. 59 (4), 259–270. 10.5603/FHC.a2021.0025 34852178

[B58] Zhang T. Chen X. Ju X. Yuan J. Zhou J. Zhang Z. (2023). PPARG is a potential target of tanshinone IIA in prostate cancer treatment: a combination study of molecular docking and dynamic simulation based on transcriptomic bioinformatics. Eur. J. Med. Res. 28, 487. 10.1186/s40001-023-01477-w 37932808 PMC10626789

[B59] Zhang Y. Y. Xie N. Sun X. D. Nice E. C. Liou Y. C. Huang C. (2024). Author correction: insights and implications of sexual dimorphism in osteoporosis. Bone Res. 12 (1), 25. 10.1038/s41413-024-00329-5 38622123 PMC11018736

[B60] Zhou J. Jiang Y. Y. Chen H. Wu Y. C. Zhang L. (2020). Tanshinone I attenuates the malignant biological properties of ovarian cancer by inducing apoptosis and autophagy *via* the inactivation of PI3K/AKT/mTOR pathway. Cell. Prolif. 53 (2), e12739. 10.1111/cpr.12739 31820522 PMC7046305

[B61] Zhou X. Lian K. Jia J. Zhao X. Duan P. Huang J. (2024). Functions of epimedin C in a zebrafish model of glucocorticoid-induced osteoporosis. J. Cell. Mol. Med. 28, e18569. 10.1111/jcmm.18569 39072972 PMC11284123

[B62] Zuo Y. Chen C. Liu F. Hu H. Dong S. Shen Q. (2024). Pinoresinol diglucoside mitigates dexamethasone-induced osteoporosis and chondrodysplasia in zebrafish. Toxicol. Appl. Pharmacol. 484, 116884. 10.1016/j.taap.2024.116884 38442791

