# Supplementary material for: Therapeutic functions of tanshinone IIA in a zebrafish model of glucocorticoid-induced osteoporosis
Source: Front Pharmacol. 2026 Mar 18;17:1772925. doi: 10.3389/fphar.2026.1772925 (PMC13038882; doi:10.3389/fphar.2026.1772925)
Supplement: Supplementary file 1 [file Supplementaryfile1.docx]

**Therapeutic Functions of Tanshinone IIA in a Zebrafish Model of Glucocorticoid-Induced Osteoporosis**

Xinyu Li ^a,b,1^, Xiaoyang Zhou ^a,b,1^, Zhiyong Li ^c^, Zilu Zhu ^d^, Songtao Wang ^b^, Jiaolong Huang ^b^, Kai Lian ^b^, Peng Duan ^b^, Chunhui Hu ^b,*^, Yihua Shi ^a,*^

^a^ Department of Orthopedics, Xiangyang No.1 People's Hospital, Hubei University of Medicine, Xiangyang 441000.

^b^ Key Laboratory of Zebrafish Modeling and Drug Screening for Human Diseases of Xiangyang City, Xiangyang No. 1 People's Hospital, Hubei University of Medicine, Xiangyang 441000.

^c^ Department of Orthopedics, Postgraduate Union training base of Xiangyang No.1 People's Hospital, School of Medicine, Wuhan University of Science and Technology, Xiangyang, 441000.

^d^ Department of Ophthalmology, Xiangyang No.1 People's Hospital, Hubei University of Medicine, Xiangyang 441000.

^⁎^ Correspondence to: Dr Chunhui Hu, Xiangyang No. 1 People's Hospital, Hubei University of Medicine, No.15 Jiefang Road, fancheng district, Xiangyang 441000, China.

E-mail address: zebrafish_team@163.com (CH. Hu).

^⁎^ Correspondence to: Dr Yihua Shi, Xiangyang No. 1 People's Hospital, Hubei University of Medicine, No.15 Jiefang Road, fancheng district, Xiangyang 441000, China.

E-mail address: yihua19@163.com (YH. Shi).

^1^ Xinyu Li and Xiaoyang Zhou are co-first authors and contributed equally to this work.

Text S1: Details of transcriptome analysis

RNA purification, reverse transcription, library construction, and sequencing were performed at BGI Tech Solutions Co., Ltd. (Wuhan, China). Library construction was performed using total RNA, with raw data quality values and other information determined, and sequencing data quality assessed using FASTQ. Differential expression analysis was similarly replicated for biologically repeated samples. The fragments per kilobase per million (FPKM) values for each gene were calculated based on gene length and the count of reads mapped to the gene. We aligned clean reads to the reference genome using Bowtie2. The raw data contained low-quality reads, adapter-contaminated reads, and reads with high N-base content. These reads required filtering prior to data analysis.

**Table S1.** The total raw reads and total clean reads for each sample.

| Sample | Total Raw Reads (M) | Total Clean Reads (M) | Total Clean Bases (Gb) | Clean Reads Q20 (%) | Clean Reads Q30 (%) | Clean Reads Ratio (%) |
| --- | --- | --- | --- | --- | --- | --- |
| Con1 | 45.44 | 44.4 | 6.66 | 99.06 | 95.65 | 97.72 |
| Con2 | 45.44 | 44.11 | 6.62 | 99.07 | 95.76 | 97.08 |
| Con3 | 45.44 | 44.21 | 6.63 | 99.09 | 95.82 | 97.3 |
| PN1 | 45.44 | 44.23 | 6.63 | 99.06 | 95.66 | 97.34 |
| PN2 | 45.44 | 44.22 | 6.63 | 99.06 | 95.64 | 97.33 |
| PN3 | 45.44 | 44.22 | 6.63 | 99.03 | 95.57 | 97.31 |
| TSN1 | 45.44 | 44.3 | 6.65 | 99.09 | 95.78 | 97.51 |
| TSN2 | 45.44 | 44.4 | 6.66 | 99.1 | 95.84 | 97.72 |
| TSN3 | 45.44 | 44.25 | 6.64 | 99.12 | 95.89 | 97.38 |

**Table S2.** List of primers used for qRT-PCR

| Species | Gene | Primer | Primer Sequence (5’−3’) | Size (bp) | Accession number |
| --- | --- | --- | --- | --- | --- |
| *Zebrafish* | *myog* | Forward | TGAAGAAGGTGAACGAGGCC | 97 | NM_131006.1 |
|  | *myog* | Reverse | ATAGCACTGCGCAGGATCTC |  |  |
|  | *acta1b* | Forward | CTCTGGTGATGGTGTGTCCC | 82 | NM_214784.2 |
|  | *acta1b* | Reverse | TCAGACCTTTCTCCGTGTGC |  |  |
|  | *cyp3a65* | Forward | CAGTGGCCAAAGACAACACG | 111 | NM_001037438.2 |
|  | *cyp3a65* | Reverse | TCAGACCTTTCTCCGTGTGC |  |  |
|  | *cyp26a1* | Forward | GACGAGCAAGAACTGGTGGA | 110 | NM_131146.2 |
|  | *cyp26a1* | Reverse | AAATTGCGTGCCCTCAAACC |  |  |
|  | *cyp7a1* | Forward | TTGCGCATGCTTTTGAACGA | 168 | NM_201173.2 |
|  | *cyp7a1* | Reverse | CTCCTCACTTGCAGCCTTCA |  |  |
|  | *col2a1b* | Forward | TCCCCTGAGGTACATGAGGG | 165 | NM_001281478.1 |
|  | *col2a1b* | Reverse | CACTGCTTCAGGTCTCTGCA |  |  |
|  | *col1a1b* | Forward | GTATTGCTGGTGCTCCTGGT | 143 | NM_201478.1 |
|  | *col1a1b* | Reverse | AGGCTCTCCCTTAGGACCAG |  |  |
|  | *rdh1* | Forward | CGGCTTTGGAAACCTTGTGG | 142 | NM_198069.1 |
|  | *rdh1* | Reverse | GTCGGTGACATTGAGCTGGA |  |  |
|  | *acana* | Forward | TCGCGTGTAAGAGTATCGGC | 143 | XM_073907260.1 |
|  | *acana* | Reverse | GCATTGGTGACGAGGAGACA |  |  |
|  | *col9a3* | Forward | CGTAGGACCCCAAGGAGAGA | 147 | NM_001305573.1 |
|  | *col9a3* | Reverse | AGTTCCCTGATGTGCTGCTC |  |  |
|  | *acaa1* | Forward | TGATGGCTCGAGTTGCACAT | 155 | NM_001002207.1 |
|  | *acaa1* | Reverse | AACACCACAAGCAAGTCCCA |  |  |
|  | *slc27a2a* | Forward | CAAACAGGTGAAGGTGCAGC | 118 | NM_001025299.1 |
|  | *slc27a2a* | Reverse | GAAGATCTCCTCCGTCAGCG |  |  |
|  | *fabp1b.1* | Forward | CAAGAGGGCTTCGTGGAGTT | 143 | NM_001024651.2 |
|  | *fabp1b.1* | Reverse | AGCACTTTTGATCCGGTCGT |  |  |
|  | *acox1* | Forward | TAGTGGGAGAGTCAGCACGA | 96 | NM_001005933.2 |
| *Zebrafish* | *acox1* | Reverse | GTTCACCTGGGCGTAGTTCA |  |  |
|  | *sod1* | Forward | CAACACAAACGGCTGCATCA | 104 | NM_131294.1 |
|  | *sod1* | Reverse | CATTACCCAGGTCTCCGACG |  |  |
|  | *jun* | Forward | ACAGCGCTTTCTCTCAGCAT | 164 | NM_199987.1 |
|  | *jun* | Reverse | TTTGAGAAGTCCCACGTCGG |  |  |
|  | *fosab* | Forward | ATCGCCAACCTGCTCAAAGA | 129 | NM_205569.1 |
|  | *fosab* | Reverse | GACTGAGATGGAGCCCATGG |  |  |
|  | *acadm* | Forward | TCAAGCTGGACTCCGGTTTC | 169 | NM_213010.2 |
|  | *acadm* | Reverse | GGCAGCTGGAACAATCTCCT |  |  |
|  | *ephx2* | Forward | TTGGGTCTCCGGATGATCCT | 164 | NM_001008642.1 |
|  | *ephx2* | Reverse | GATCTTCGCTCTCGGTCTGG |  |  |
|  | *keap1a* | Forward | GGCATCTCGAGATTCGCTGA | 168 | NM_182864.2 |
|  | *keap1a* | Reverse | GCAGAGCACCTTCAGACTGT |  |  |
|  | *akt2l* | Forward | GCCTCTGGGATGTACTACGC | 87 | NM_212815.2 |
|  | *akt2l* | Reverse | ACTCTCTGTGACTGTGTGCG |  |  |
|  | *kras* | Forward | TCGAATGCACGCTACGCTAT | 167 | NM_001003744.2 |
|  | *kras* | Reverse | TTGAACTTTCCAACGCAGCG |  |  |
|  | *ddit3* | Forward | ACACTCGGTGACTTCAGCTG | 89 | NM_001082825.1 |
|  | *ddit3* | Reverse | GGCTGCTTCTGATTCAGGGT |  |  |
|  | *cyp2p7* | Forward | ACAGGGTGACAATCTGGCTG | 167 | NM_001083049.1 |
|  | *cyp2p7* | Reverse | TGGACGGCTCCAGACTTTTC |  |  |
|  | *cyp2p8* | Forward | CACTTCCTGGATGCTGAGGG | 85 | NM_001083035.2 |
|  | *cyp2p8* | Reverse | GCTCCCCAAGACACACTCTC |  |  |
|  | *Gapdh* | Forward | TCCGTCTTGAGAAACCTGCC | 105 | NM_001115114.1 |
|  | *Gapdh* | Reverse | CAACCTGGTGCTCCGTGTAT |  |  |


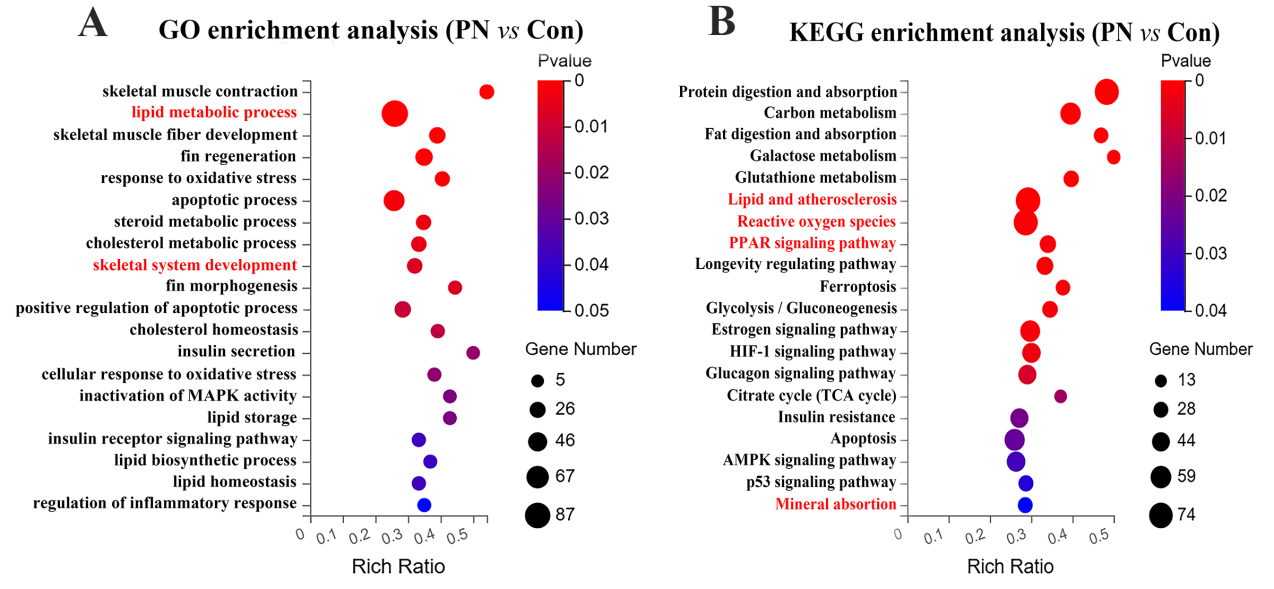


**Fig. S1. Transcriptome Analysis of zebrafish Larvae exposed to 25 μmol/L PN for 5 d.** (A) GO enrichment analysis of upregulated and downregulated DEGs. (B) KEGG enrichment of upregulated and downregulated DEGs.


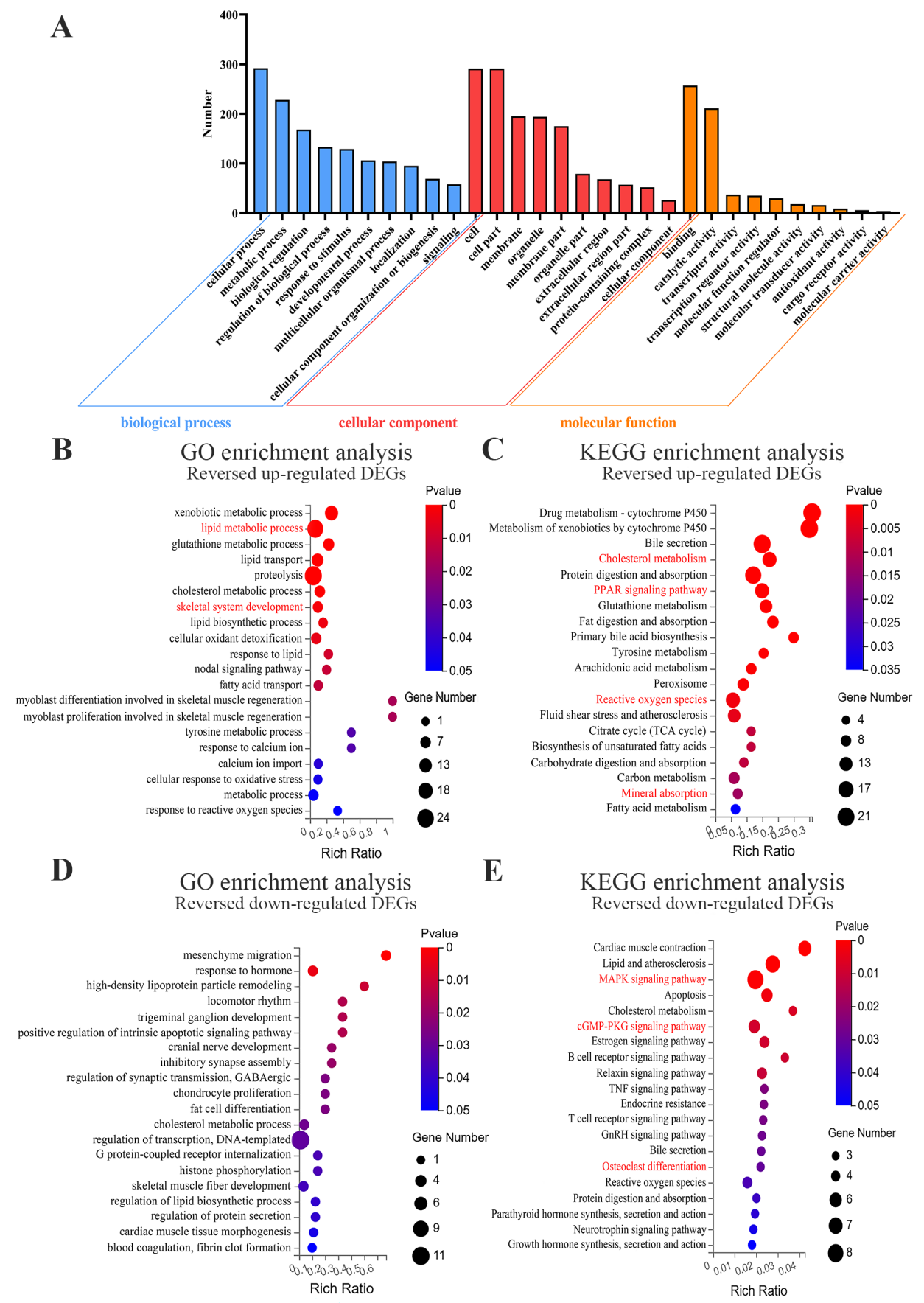


**Fig. S2. GO and KEGG enrichment of reversed differentially expressed genes.** (A) GO enrichment analysis of reversed DEGs**.** (B) GO enrichment analysis of the reversed upregulated DEGs. (C) KEGG enrichment of the reversed upregulated DEGs. (D) GO enrichment analysis of the reversed downregulated DEGs. (E) KEGG enrichment analysis of the reversed downregulated DEGs.
